# Adjuvant Therapy after Esophagectomy for Esophageal Cancer: Who Needs It?: Multi-institution Worldwide Observational Study

**DOI:** 10.1097/AS9.0000000000000497

**Published:** 2024-10-15

**Authors:** Siva Raja, Thomas W. Rice, Min Lu, Marie E. Semple, Andrew J. Toth, Eugene H. Blackstone, Sudish C. Murthy, Usman Ahmad, Michael McNamara, Hemant Ishwaran

**Affiliations:** From the * Department of Thoracic and Cardiovascular Surgery, Heart, Vascular, and Thoracic Institute, Cleveland Clinic, Cleveland, OH; † Division of Biostatistics, Department of Public Health Sciences, University of Miami, Miami, FL; ‡ Lerner Research Institute, Department of Quantitative Health Sciences, Cleveland Clinic, Cleveland, OH; § Department of Hematology and Medical Oncology, Taussig Cancer Institute, Cleveland Clinic, Cleveland, OH.

**Keywords:** esophagectomy, esophageal and esophagogastric cancer, random forest analysis, virtual-twin analysis

## Abstract

**Objective:**

Based on current practice guidelines, we hypothesized that most patients with esophageal cancer, particularly those with locally advanced cancer, would benefit from adjuvant therapy after esophagectomy *versus* esophagectomy alone. We sought to obtain a granular estimate of patient-level risk-adjusted survival for each therapeutic option by cancer histopathology and stage.

**Background:**

Although esophagectomy alone is now an uncommon therapy for treating locally advanced esophageal cancer, the value of adjuvant therapy after esophagectomy is unknown.

**Methods:**

From 1970 to 2014, 22,123 consecutive patients from 33 centers on 6 continents (Worldwide Esophageal Cancer Collaboration) were diagnosed with biopsy-proven adenocarcinoma (n = 7526) or squamous cell carcinoma (n = 5625), of whom 10,873 received esophagectomy alone and 2278 additional adjuvant therapy. Random forests for survival and virtual-twin analyses were performed for all-cause mortality.

**Results:**

For adenocarcinoma, adjuvant therapy was beneficial only in pT4NanyM0 cancers (6–8 month survival benefit) and in pTanyN3M0 cancers (4–8 month benefit); a survival decrement was observed in pT1-3N0M0 cancers, with no effect on TanyN1-2M0 cancers. In squamous cell carcinoma, there was a 4 to 21 month survival benefit for pT3-4N0M0 cancers and a 4 to 15 month survival benefit for pT2-4N1-3M0 cancers.

**Conclusions:**

Adjuvant therapy after esophagectomy appears to benefit most patients with node-positive squamous cell carcinoma, but for adenocarcinoma, its value is limited to deep cancers and to those with substantial nodal burden. Future studies of the role of adjuvant therapies should treat these 2 cancers differently, with guidelines reflecting the histopathologic-appropriate survival value of adjuvant therapy.

## INTRODUCTION

Current guidelines for using adjuvant therapy based on pathologic stage mirror the guidelines for neoadjuvant therapy using a clinical stage. The role of adjuvant therapy after esophagectomy alone for esophageal cancer is unknown because it has been difficult to conduct appropriate studies given the ubiquitous use of neoadjuvant therapy in many stage classifications. An understanding of adjuvant therapy’s role will be helpful in determining its benefit for future patients undergoing esophagectomy or endoscopic resection as initial therapy, and in providing a baseline for comparing current and future therapies.

Thus, in this study, we present our analysis of patients who did or did not receive adjuvant therapy after esophagectomy alone using the granular 6-continent Worldwide Esophageal Cancer Collaboration (WECC) database,^1^ which was used to develop edition 8 cancer staging manuals.^2,3^ Based on current National Comprehensive Cancer Network guidelines, we hypothesized that any patient with esophageal cancer, particularly those with locally advanced cancer, would benefit from adjuvant therapy after esophagectomy (Ajani JA, D’Amico TA, Bentrem DJ, et al. Esophageal and esophagogastric junction cancers, version 4.2022, NCCN Clinical Practice Guidelines in Oncology, as yet unpublished). Given the global nature of our database, the intent of this study was to examine the value of adjuvant therapy after esophagectomy alone generally, not to examine the effectiveness of any specific protocol.

In contrast to studies that have used propensity-score methods to estimate average effects on survival of these 2 treatment strategies,^4–6^ we used virtual-twin analysis, introduced in 2011 by Foster et al,^7^ to assess survival on an individual patient basis. As with propensity-score methods,^8^ we first identified patients likely to receive either therapy, which would represent real-world “virtual equipoise” or “overlap.” Then, to compare survival with both therapies, with each patient serving as his or her own control (“virtual twin”), we calculated risk-adjusted predicted survival for that individual patient with therapy received and again with therapy not received (counterfactual).

Thus, the objective of our study was to determine the individual benefit for patients within different cancer categories while accounting for the interplay of pT and pN categories for those undergoing adjuvant therapy after esophagectomy alone for adenocarcinoma and squamous cell carcinoma of the esophagus or esophagogastric junction.

## PATIENTS AND METHODS

### Patients

From 1970 to 2014 at 33 WECC institutions (Supplemental Table S1, http://links.lww.com/AOSO/A406, listing participating institutions and investigators), 22,123 consecutive patients had real-world clinical staging data available for epithelial cancers of the esophagus and esophagogastric junction as part of the effort to provide clinical^1,9^ and pathologic^10,11^ staging data for the 8th edition cancer staging manuals.^2,3^ Of these, 13,151 had adenocarcinoma or squamous cell carcinoma and underwent esophagectomy alone (adenocarcinoma n = 6560, squamous cell carcinoma n = 4313) or esophagectomy followed by adjuvant therapy (adenocarcinoma n = 966; squamous cell carcinoma n = 1312) (Supplemental Table S2, http://links.lww.com/AOSO/A406, showing baseline and treatment data stratified by therapy received).

### Adjuvant Therapies

Of the 966 patients with adenocarcinoma receiving adjuvant therapy after esophagectomy, 403 received chemotherapy only, 128 radiotherapy only, and 406 chemoradiotherapy (therapy details unknown in 29). Of the 1312 patients with squamous cell carcinoma receiving adjuvant therapy after esophagectomy, 646 received chemotherapy only, 253 radiotherapy only, and 413 chemoradiotherapy (Supplemental Table S3, http://links.lww.com/AOSO/A406, showing baseline and treatment data stratified by histopathologic cell type).

### Data

This study used 36 variables representing patient demographics, comorbidities, and cancer characteristics (Supplemental Appendix S1, http://links.lww.com/AOSO/A406 listing variables used in random forest analyses), cancer treatment, and time-related mortality, with site and continent excluded to contain data dimensionality and reduce confounding with treatment.^1^ These variables were obtained after the local ethics board approval of databases and data-use agreements with Cleveland Clinic. Data were requested in deidentified or anonymized form using standard definitions. The Case Cancer Institutional Review Board of Case Western Reserve University and the Cleveland Clinic Institutional Review Board approved the entire project and use of these data for research, with patient consent waived.

Missing data for independent variables were imputed using “on-the-fly” random forest imputation^12^ implemented in the open-source randomForestSRC R-software package^13^ under default settings.

### Endpoint

The endpoint was all-cause mortality from the first management decision after esophageal cancer diagnosis. Among all patients with adenocarcinoma or squamous cell carcinoma undergoing esophagectomy with or without adjuvant therapy, the median potential follow-up^14,15^ was 10.8 years had there been no deaths, but considering deaths in this elderly population with a rapidly lethal cancer, median observed follow-up was 1.8 years. For patients receiving esophagectomy only, 50% were followed >1.9 years, 25% >4.4 years, and 10% >7.8 years; for those receiving neoadjuvant therapy plus adjuvant therapy, 50% were followed >1.5 years, 25% >2.8 years, and 10% >4.5 years.

### Statistical Analysis

Analyses were conducted separately for adenocarcinoma and squamous cell carcinoma.^2,3^ For these cancers, the initial therapy decision is whether or not to perform esophagectomy only. The subsequent addition of adjuvant therapy is a decision made after the pathologic classification of the cancer (pTNM) is determined. Adjuvant therapy after esophagectomy is, therefore, the focus of this study. The primary objective of the statistical analysis is to identify patients for whom addition of adjuvant therapy to esophagectomy is predicted to be beneficial, makes no difference, or is harmful based on differences in survival time. This was accomplished in 4 analytic steps:

1. *Eligibility:* We identified patients deemed eligible for both strategies based on observed clinical practice across the world [Supplemental Appendix S2, http://links.lww.com/AOSO/A406, describing the method for determining the probability of patient eligibility (overlap)].^16^ Although theoretically, all patients in this study could have received esophagectomy with or without adjuvant therapy, esophagectomy alone was nearly always used for early-stage cancers and adjuvant therapy after esophagectomy for deeply invasive, node-positive cancers. Comparison of survival for these patients was considered unfair, and therefore they were excluded from further analysis (Supplemental Table S4, http://links.lww.com/AOSO/A406, describing characteristics of patients with adenocarcinoma deemed eligible or not for both therapies, and Supplemental Table S5, http://links.lww.com/AOSO/A406, describing characteristics of such patients with squamous cell carcinoma). Those deemed eligible to receive either therapy were included in the final analysis. This data-driven strategy is similar to that used in propensity-score-based analyses.^8^
2. *Survival analysis:* We performed multivariable survival analyses for patients found eligible for both esophagectomy alone and adjuvant therapy after esophagectomy. The analysis was performed using random survival forests virtual-twin interaction (RSF-VT-I), which incorporated interaction terms for all clinical and cancer variables with the 2 treatment strategies (Supplemental Appendix S3, http://links.lww.com/AOSO/A406, describing survival analysis).^13,17^ This differs from comparisons based on propensity-matched pairs of patients because virtual twins are, by definition, exact matches.^7,18^
3. *Virtual-twin analysis:* From the survival analysis, we generated 2 predicted survival curves for each patient, 1 for actual treatment received and 1 for the counterfactual treatment.^7,17,18^ This was accomplished using the identical patient data, but substituting the counterfactual therapy for the actual therapy received.
4. *Gain or loss of lifetime:* We calculated the area under each pair of survival curves for each patient from initial therapy to 10 years—a measure of length of life within those 10 years called restricted mean survival time—and took the difference.^19,20^ Differences in restricted mean survival time between survival curves for pairs of therapies estimated the amount of survival time gained (positive number) or lost (negative number) by being treated with esophagectomy alone or with adjuvant therapy after esophagectomy. From this difference, we identified the cancer profile of those benefiting or not benefiting from adjuvant therapy.

## RESULTS

### Therapy Eligibility (Virtual Equipoise)

#### Adenocarcinoma

Among patients with adenocarcinoma, 3703 (49% of 7526) were deemed eligible for esophagectomy with or without adjuvant therapy (Table 1). They constituted the study group for adenocarcinoma (Supplemental Table S6, http://links.lww.com/AOSO/A406, showing baseline characteristics of patients deemed eligible for both esophagectomy alone or combined with adjuvant therapy, stratified by histopathologic cell type). Excluded were 3823 patients (51% of 7526) who did not have a reasonable chance of receiving both therapies (see Supplemental Table S5, http://links.lww.com/AOSO/A406).

**TABLE 1.** Cancer Characteristics of Patients Deemed Eligible for Esophagectomy Alone or Combined With Adjuvant Therapy, Stratified by Pure Histopathologic Cell Type

	Esophagectomy Alone				Adjuvant Therapy after Esophagectomy			
	Adenocarcinoma (N = 2817)		Squamous Cell Carcinoma (N = 2558)		Adenocarcinoma (N = 886)		Squamous Cell Carcinoma (N = 1229)	
Cancer Characteristics	n^*^	No. (%)	n^*^	No. (%)	n^*^	No. (%)	n^*^	No. (%)
Pathologic T	2803		2557		881		1228	
Tis		34 (1.2)		62 (2.4)		0 (0)		5 (0.41)
1		277 (9.9)		373 (15)		57 (6.5)		111 (9.0)
2		425 (15)		462 (18)		102 (12)		188 (15)
3		1914 (68)		1305 (51)		649 (74)		716 (58)
4		153 (5.5)		355 (14)		73 (8.3)		208 (17)
X		14		1		5		1
Pathologic N	2557		2469		799		1202	
0		659 (26)		1301 (53)		109 (14)		536 (45)
1		656 (26)		642 (26)		183 (23)		347 (29)
2		643 (25)		394 (16)		223 (28)		229 (19)
3		599 (23)		132 (5.3)		284 (36)		90 (7.5)
X		260		89		87		27
Pathologic M1	2817	384 (14)	2558	158 (6.2)	886	147 (17)	1229	48 (3.9)
Resection margin	2817		2558		886		1229	
0		2360 (84)		2324 (91)		734 (83)		1163 (95)
1		350 (12)		187 (7.3)		128 (14)		42 (3.4)
2		107 (3.8)		47 (1.8)		24 (2.7)		24 (2.0)

* Patients with data available.

As an example, within the group of patients with esophageal adenocarcinoma deemed to have a low probability of receiving adjuvant therapy after esophagectomy, only 36 of 3775 (0.95%) actually received it. Similarly, within the group deemed to have a high probability of receiving adjuvant therapy after esophagectomy, 44 of 48 (92%) actually received it (see Supplemental Table S5, http://links.lww.com/AOSO/A406).

#### Squamous Cell Carcinoma

Among patients with squamous cell carcinoma, 3787 (67% of 5625) were deemed eligible for esophagectomy with or without adjuvant therapy (Table 1). They constituted the study group for squamous cell carcinoma (see Supplemental Table, http://links.lww.com/AOSO/A406, showing baseline characteristics of patients deemed eligible for both esophagectomy alone or combined with adjuvant therapy, stratified by histopathologic cell type). Excluded were 1838 patients (33% of 5625) who did not have a reasonable chance of receiving both therapies (see Supplemental Table S5, http://links.lww.com/AOSO/A406).

As an example, within the group of patients with esophageal squamous cell carcinoma deemed to have a low probability of receiving adjuvant therapy after esophagectomy, only 25 of 1778 (1.4%) actually received it. Similarly, within the group deemed to have a high probability of receiving adjuvant therapy after esophagectomy, 58 of 60 (97%) actually received it (see Supplemental Table S5, http://links.lww.com/AOSO/A406).

### Survival

#### Adenocarcinoma

**FIGURE 1.**
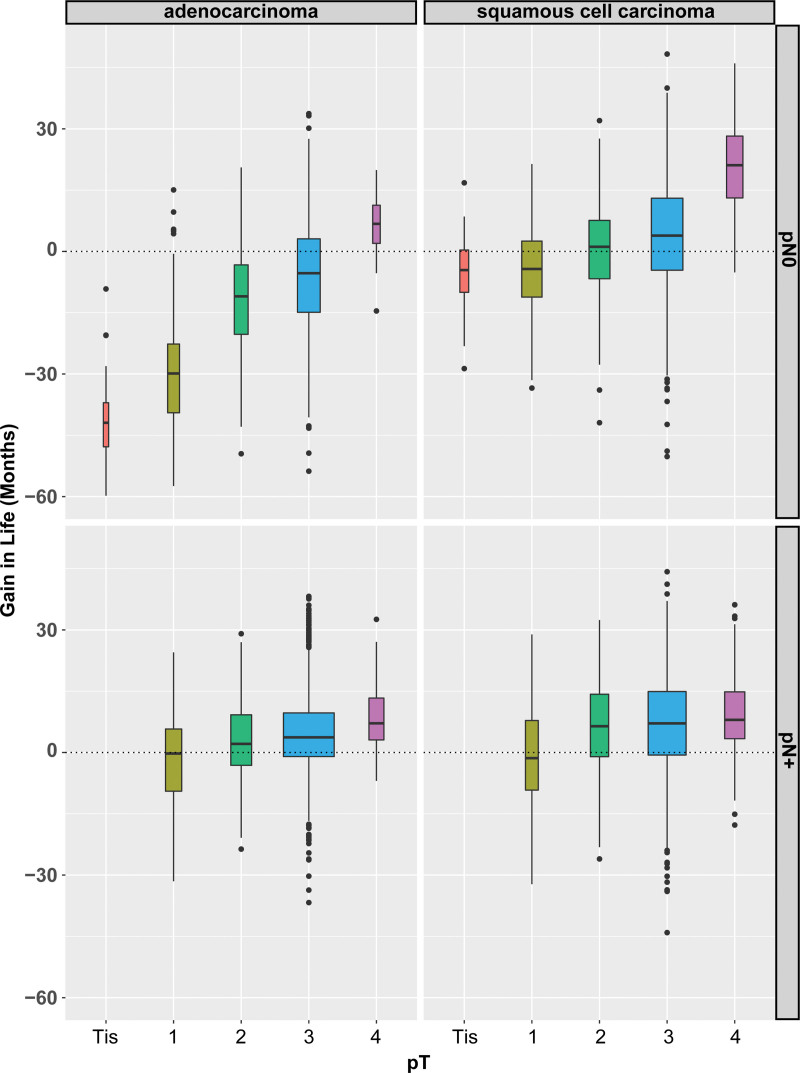
Box and whiskers plot of gain (positive) or detriment (negative) in lifetime within 10 years by adding adjuvant therapy after esophagectomy according to pT category along the horizontal axis, and pN0 and pN+ along the right-hand edge for adenocarcinoma (left) and squamous cell carcinoma (right). Solid horizontal bar is median, the box encloses the 25th and 75th percentiles of values, whiskers are 1.5 times the interquartile range, and filled circles are values beyond this. Box width is proportional to sample size. When the median (solid bar) is above zero, there is a gain in lifetime, and, when below zero, a detriment in lifetime, for that pT category.

**FIGURE 2.**
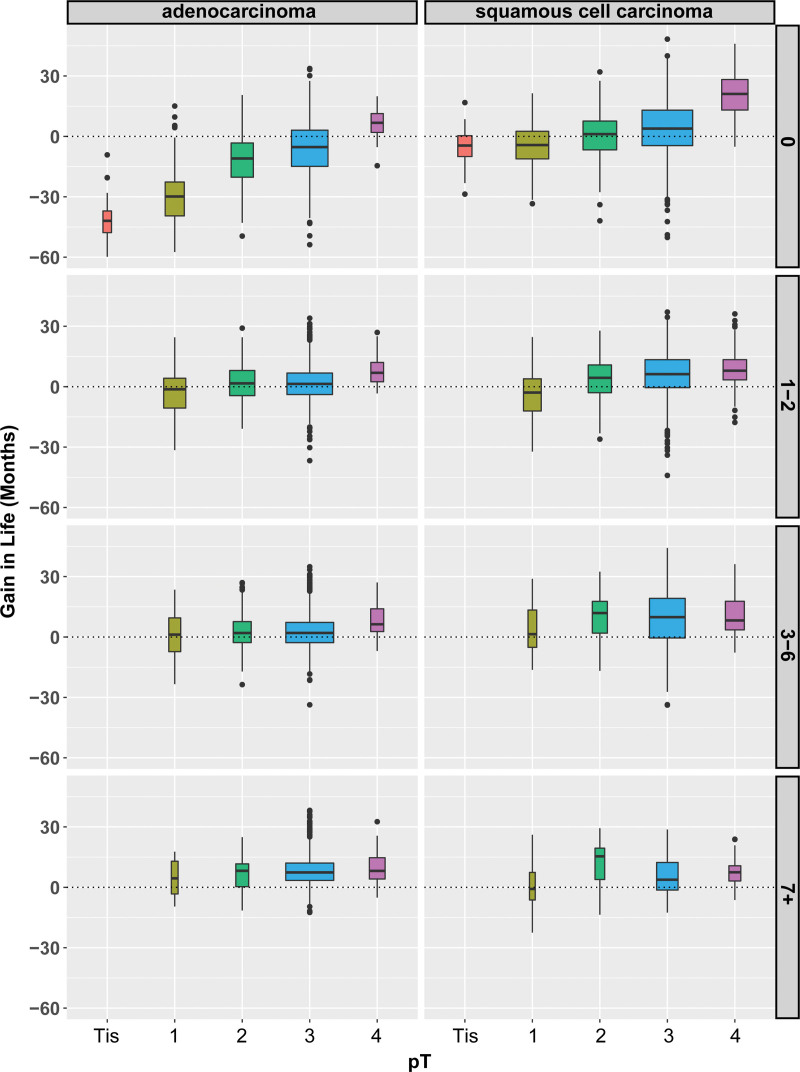
Box and whiskers plot of gain (positive) or detriment (negative) in lifetime within 10 years by adding adjuvant therapy after esophagectomy according to pT category along the horizontal axis, and number of cancer-positive lymph nodes (right-hand edge) for adenocarcinoma (left) and squamous cell carcinoma (right). Format is as in Figure 1.

For patients with adenocarcinoma who were node-negative (N0), the only subset benefiting from adjuvant therapy after esophagectomy was patients with pT4 adenocarcinoma; the remainder had varying degrees of survival detriment, with the detriment increasing with decreasing pT category. Patients with node-positive adenocarcinoma undergoing adjuvant therapy with a pT2-4 depth of invasion had a survival benefit (Fig. 1). In a more granular analysis, for patients with pT1-3 adenocarcinomas, a meaningful benefit was seen only in pN3 adenocarcinoma (Fig. 2 and Table 2). For pN1-2, there was no meaningful benefit except for pT4 adenocarcinoma.

**TABLE 2.** Median Months of Life Gained (Positive Values) or Lost (Negative Values) From Adjuvant Therapy After Esophagectomy for Adenocarcinoma or Squamous Cell Cancer of the Esophagus, and 5-year Survival Without (Eso) and With (Eso+) Adjuvant Therapy After Esophagectomy

Depth of Invasion (T)	pN0			pN+			pN1			pN2			pN3		
		Survival (%)			Survival (%)			Survival (%)			Survival (%)			Survival (%)	
	RMS	Eso	Eso+	RMS	Eso	Eso+	RMS	Eso	Eso+	RMS	Eso	Eso+	RMS	Eso	Eso+
Adenocarcinoma															
pTis	−48	76	29												
pT1	−30	71	41	−0.3	40	38	−1.3	45	42	1.2	28	29	4.5	20	22
pT2	−11	53	41	2.1	26	28	1.7	32	33	2.0	21	24	8.2	11	16
pT3	−5.3	39	33	3.7	13	16	1.4	20	21	2.0	14	16	7.4	5	11
pT4	6.8	13	18	7.2	7	13	6.9	6	7	6.3	7	13	8.2	1	3
Squamous cell carcinoma															
pTis	−4.6	69	62												
pT1	−4.3	62	53	−1.4	37	33	−2.9	41	36	1.4	27	30	−0.8	16	16
pT2	1.1	52	48	6.4	24	29	4.4	29	31	12	19	28	15.4	11	20
pT3	3.9	42	43	7.1	17	22	6.2	21	25	9.9	14	21	3.8	7	11
pT4	21	16	32	8.0	13	20	8.0	16	22	8.2	10	19	7.5	6	11

Red shading denotes detriment, green insignificant benefit, and blue meaningful benefit.

Eso indicates esophagectomy alone; Eso+, esophagectomy followed by adjuvant therapy; RMS, restricted mean survival difference.

As an example, for pT2N1M0 adenocarcinoma, patients had a 1.7-month survival benefit, while those with pT2N3M0 adenocarcinoma had an 8.2-month survival benefit (Table 2). Patients with pT2N0M0 adenocarcinoma had an 11-month survival detriment with the addition of adjuvant therapy over esophagectomy alone.

#### Squamous Cell Carcinoma

The pattern of gain or loss in lifetime was different for squamous cell carcinoma. Adjuvant therapy after esophagectomy had a survival benefit for most patients with deep node-negative and any node-positive squamous cell cancer (Fig. 1). Among those with node-negative carcinoma, only patients with pT3-4 cancers had a significant survival benefit from adjuvant therapy. Varying degrees of detriment with the addition of adjuvant therapy for node-negative cancers was seen with decreasing T category among pT1-2 squamous cell cancers. The incremental survival detriment was less than that for similar T categories in adenocarcinoma. For node-positive squamous cell carcinoma, there was a survival benefit of adjuvant therapy for pT2-4 squamous cell cancers (Fig. 2 and Table 2). The incremental benefit in survival for pT2-3 was greater than for their adenocarcinoma counterparts. In a granular analysis of node-positive cancers, there was a benefit of adjuvant therapy for pT2-4N1-3M0 squamous cell carcinomas, unlike adenocarcinoma, for which the benefit was limited to pT4NanyM0 and pTanyN3M0.

As an example, patients with pT2N1M0 had a 4.4-month survival benefit from adjuvant therapy, and those with pT2N3M0 had a 15-month benefit (Table 2). In contrast, those with pT1N0M0 had a 4.3-month survival detriment from adjuvant therapy. In comparing adenocarcinoma to squamous cell carcinoma, patients with pT3N1M0 adenocarcinoma had only a 1.4-month survival benefit with adjuvant therapy *versus* a 6.2-month benefit for a similarly staged squamous cell carcinoma.

## DISCUSSION

### Principal Findings

Considering all patients in our study, adjuvant therapy after esophagectomy offered a survival benefit primarily for patients with node-positive cancer. There appeared to be a survival detriment in patients with node-negative cancer (pN0) unless it was deeply invasive. For adenocarcinoma, adjuvant therapy had a meaningful survival benefit only for pT4 or pN3 cancers. For squamous cell carcinoma, adjuvant therapy had a meaningful survival benefit for pT3-4N0M0 cancers and pT2-4N1-3M0 cancers. In general, adjuvant therapy was more effective in patients with squamous cell cancer than those with adenocarcinoma.

### Context and Literature Review

The National Comprehensive Cancer Network guidelines recommend adjuvant therapy after esophagectomy for patients with deeper cancers (T2 or greater) and node-positive disease (N1-3) in adenocarcinoma, and no adjuvant therapy after esophagectomy regardless of stage in R0 resection for patients with squamous cell cancer (Ajani JA, D’Amico TA, Bentrem DJ, et al. Esophageal and esophagogastric junction cancers, version 4.2022, NCCN clinical practice guidelines in oncology, as yet unpublished). Our first analysis seems to support the use of adjuvant therapy for all node-positive and deeper esophageal cancers, irrespective of histopathologic cell type. However, a more granular analysis suggests that the survival benefit is derived from patients with greater nodal involvement. There does not appear to be a significant benefit of adjuvant therapy for pN1-2 adenocarcinomas that have been completely resected (R0) unless it is pT4, contrary to the current guideline. Conversely, our study suggests a meaningful benefit in many subgroups of patients with squamous cell cancer, contrary to the guideline.

In a prior study from our group, for patients with locoregionally advanced esophageal carcinoma, adding postoperative adjuvant chemoradiotherapy to esophagectomy alone doubled survival time, time to recurrence, and recurrence-free survival.^21^ This was 1 of the few studies showing a benefit of adjuvant therapy in patients with adenocarcinoma, but the study was too small (n = 31) to perform subgroup analysis. Macdonald et al^22^ reported a large trial investigating the value of adjuvant chemoradiotherapy in patients with gastroesophageal junction and gastric adenocarcinoma who were randomized to esophagectomy only or esophagectomy with adjuvant chemoradiotherapy. It demonstrated an increase in median survival from 27 to 36 months. However, the trial included patients in stages IB to IVA (M0), and the investigators were unable to identify subgroups that may have benefited the most. A propensity-matched analysis of squamous cell carcinoma from the Taiwan Cancer Registry revealed that adjuvant chemoradiotherapy after esophagectomy (679 without and 416 with adjuvant therapy) provided a survival benefit in cases of pT3-4pN+ cancers, cancer length >3.2 cm, poor differentiation, and positive margins.^6^

However, studies have not universally shown the benefit of adjuvant therapy after esophagectomy. A 30-center European multi-institutional propensity-matched retrospective analysis of 2944 patients with node-positive disease (178 receiving adjuvant therapy) found that adjuvant chemoradiotherapy was not associated with better survival.^23^ The recommendation for the use of adjuvant therapy after esophagectomy is currently based on these and similar small retrospective studies, with only a few able to define subsets benefiting from additional adjuvant therapy.

In the case of node-negative esophageal cancer, the value of adjuvant therapy remains unclear. In the study by Deng et al,^24^ patients with node-negative esophageal squamous cell cancer (T2-T4) realized a survival benefit from adjuvant therapy. However, most patients (two-thirds) had T3 or T4 cancers, the outcomes of which likely drove their overall findings, as noted in our study. Similarly, in the study by Rucker et al^4^ of patients with esophageal adenocarcinoma, they found no value of adjuvant therapy in node-negative patients. Given that they only had 2 patients with T4 cancer, they missed observing the value of adjuvant therapy demonstrated in this study for that T classification.

Our study is the first large-scale risk-adjusted analysis providing insight into subgroups that may benefit from adjuvant therapy after esophagectomy. It is clear that in this group, squamous cell carcinoma and adenocarcinoma need to be treated with separate algorithms. For adenocarcinoma, adjuvant therapy in the face of limited nodal involvement and complete resection did not improve survival, albeit a detriment to survival was not noted, as in the case of adjuvant therapy in most node-negative patients.

The future of esophagogastric cancer prognostication may become individualized to the patient rather than relying on population-based staging algorithms. Precision-care analysis is the individualization of treatment decisions to improve outcomes, even among apparently homogeneous populations. A recent study from this same WECC database has shown that such a survival analysis, which incorporated patient and cancer characteristics, identified an optimal treatment that potentially could have improved survival by an average of 7% over the actual therapy received.^25^ As such, our future understanding of prognostication will likely need to transcend the “classic” variables of T, N, and M.

The role of immunotherapy in the adjuvant setting is currently standard of care in patients with persistent residual disease after neoadjuvant therapy followed by esophagectomy.^26^ Its value in patients who have not received neoadjuvant therapy has yet to be defined in clinical studies.

### Strengths and Limitations

A key strength of this study is the quality of the data, which was heavily audited to ensure accuracy and completeness as data were collected to create the 8th edition cancer staging manuals.^2,3^ Another obvious strength is the large number of patients compared with previous studies. The analytic strategy is unique and a principal strength. The virtual-twin model was used to generate a survival curve for each therapy (with and without adjuvant therapy) and for each patient, allowing us to estimate gain or loss of lifetime for even the smallest of subsets within this cohort of patients. Although it has become customary to approach an analysis such as this using propensity-score methods,^8^ the biologically plausible interplay of depth of tumor invasion (T) and nodal involvement (N) due to the unique lymphatic drainage of the esophagus led us to use nonparametric random forest methods that account for the interplay of T and N.^27–30^ Propensity-score methods focus on average treatment effect on the treated, unlike the virtual-twin model, which is more informative in yielding individual treatment effects.^7,17^ Nevertheless, just as with propensity-score comparisons, virtual-twin comparisons require that a patient must be eligible for each therapy being compared. Thus, those with early-stage cancers (pT1-2N0M0, for example) were excluded from the analysis because almost all received esophagectomy alone without adjuvant therapy.

In regard to limitations, the data used in this analysis were obtained from observational institutional databases worldwide and thus include practice pattern variability. Esophageal cancer is a rare disease, and patients submitted to WECC from around the world including those treated as far back as 1970. However, the majority of patients submitted and deemed eligible for comparison of survival were treated from 2000 to 2013 (see Supplemental Figure S1, http://links.lww.com/AOSO/A406, which shows the distribution of dates of starting treatment among patients deemed eligible for both neoadjuvant therapy only and neoadjuvant therapy and adjuvant therapy). The WECC database does not contain specifics on various chemotherapy, radiotherapy, and chemoradiotherapy regimens and doses. However, the goal of this study was to examine the overall value of adjuvant therapy after esophagectomy in a general real-world setting, not to examine the effectiveness of specific protocols. The endpoint was all-cause mortality for this rapidly lethal disease. This likely included a few noncancer deaths, but it is a reliable endpoint when adjusted for patient demographics and comorbidities^31,32^ and is the basis for most cancer staging.^33,34^ We did not have morbidity information to further evaluate treatment toxicity, although hospital mortality was low.^1^ The inherent limitations of clinical staging in determining upstaging and downstaging are affected by local clinical and pathologic staging protocols among institutions.

### Conclusions and Relevance

Adjuvant therapy after esophagectomy benefits most patients who have node-positive squamous cell carcinomas (pTanyN1-3M0). Its value in adenocarcinoma appears to be limited primarily to those with pT4NanyM0 or pTanypN3M0 adenocarcinomas. Future studies should aim to confirm this new finding so guidelines can be changed to reflect the appropriate value of adjuvant therapy for individual patients according to histopathology. A better understanding of adjuvant therapy’s role will be helpful in determining its benefit for future patients undergoing esophagectomy as initial therapy and in providing a baseline for comparison with present and future therapies. The role of new therapies in the precision treatment of esophageal cancer, such as immunotherapy, is yet to be established in this group of patients who are currently being treated with curative intent. However, analogs from other malignancies make us hopeful that additional therapeutic options, including immunotherapy, will be available in the future.

## ACKNOWLEDGMENTS

S.R.: substantial contributions to the conception, design, analysis, and interpretation of the data. Also heavily involved in drafting and revising the manuscript. T.W.R.: substantial contributions to the conception, design, data collection, and interpretation of the data. Also involved in revising the manuscript. M.L. and H.I.: substantial contributions to the conception, design, and selection of statistical approach to the data, statistical analysis, and interpretation of the statistical results. Also involved in drafting and revising the manuscript. M.E.S.: substantial contributions to the design, data collection, analysis, and interpretation of the data. Also involved in drafting and revising the manuscript. A.J.T.: substantial contributions to the statistical analysis, interpretation of the data, and verifying the data in the revised manuscript. E.H.B.: substantial contributions to the conception, design, analysis, data collection, and interpretation of the data. Also heavily involved in drafting and revising the manuscript. S.C.M.: substantial contributions to the conception, design, and interpretation of the data. Also involved in revising the manuscript. U.A. and M.McN: substantial contributions to the conception, design, and interpretation of the data. Also involved in revising the manuscript. All authors have seen and given their final approval of the version being submitted for review.

## Supplementary Material

## References

[1] Rice TW Apperson-Hansen C DiPaola LM. Worldwide Esophageal Cancer Collaboration: clinical staging data. Dis Esophagus. 2016;29:707–714. 27731549 10.1111/dote.12493 PMC5591441

[2] *American Joint Committee on Cancer*. Cancer Staging Manual. 8 ed. New York, NY: Springer; 2017.

[3] Brierley JD Gospodarowicz MK Witte C. TNM Classification of Malignant Tumours. 8th ed. New York, NY: Wiley.

[4] Rucker AJ Raman V Jawitz OK. The impact of adjuvant therapy on survival after esophagectomy for node-negative esophageal adenocarcinoma. Ann Surg. 2022;275:348–355. 32209899 10.1097/SLA.0000000000003886 PMC7502525

[5] Zhu K Ren P Yang Y. Role of chemotherapy after curative esophagectomy in squamous cell carcinoma of the thoracic esophagus: a propensity score-matched analysis. Thorac Cancer. 2021;12:1800–1809. 33943011 10.1111/1759-7714.13981 PMC8201545

[6] Hwang JY Chen HS Hsu PK. A propensity-matched analysis comparing survival after esophagectomy followed by adjuvant chemoradiation to surgery alone for esophageal squamous cell carcinoma. Ann Surg. 2016;264:100–106. 26649580 10.1097/SLA.0000000000001410

[7] Foster JC Taylor JM Ruberg SJ. Subgroup identification from randomized clinical trial data. Stat Med. 2011;30:2867–2880. 21815180 10.1002/sim.4322 PMC3880775

[8] Rubin DB. The design versus the analysis of observational studies for causal effects: parallels with the design of randomized trials. Stat Med. 2007;26:20–36. 17072897 10.1002/sim.2739

[9] Rice TW Ishwaran H Blackstone EH; Worldwide Esophageal Cancer Collaboration Investigators. Recommendations for clinical staging (cTNM) of cancer of the esophagus and esophagogastric junction for the 8th edition AJCC/UICC staging manuals. Dis Esophagus. 2016;29:913–919. 27905171 10.1111/dote.12540 PMC5591442

[10] Rice TW Chen LQ Hofstetter WL. Worldwide esophageal cancer collaboration: pathologic staging data. Dis Esophagus. 2016;29:724–733. 27731547 10.1111/dote.12520 PMC5731491

[11] Rice TW Ishwaran H Hofstetter WL; Worldwide Esophageal Cancer Collaboration Investigators. Recommendations for pathologic staging (pTNM) of cancer of the esophagus and esophagogastric junction for the 8th edition AJCC/UICC staging manuals. Dis Esophagus. 2016;29:897–905. 27905172 10.1111/dote.12533 PMC5591444

[12] Tang F Ishwaran H. Random forest missing data algorithms. Stat Anal Data Min. 2017;10:363–377. 29403567 10.1002/sam.11348 PMC5796790

[13] Ishwaran H Kogalur UB. Random forests for survival, regression and classification (RF-SRC), R package version 2.9.3. Available at: http://cran.r-project.org/web/packages/randomForestSRC/index.html; 2020.

[14] Korn EL. Censoring distributions as a measure of follow-up in survival analysis. Stat Med. 1986;5:255–260. 3738291 10.1002/sim.4780050306

[15] Schemper M Smith TL. A note on quantifying follow-up in studies of failure time. Control Clin Trials. 1996;17:343–346. 8889347 10.1016/0197-2456(96)00075-x

[16] O’Brien R Ishwaran H. A random forests quantile classifier for class imbalanced data. Pattern Recognit. 2019;90:232–249. 30765897 10.1016/j.patcog.2019.01.036 PMC6370055

[17] Lu M Sadiq S Feaster DJ. Estimating individual treatment effect in observational data using random forest methods. J Comput Graph Stat. 2018;27:209–219. 29706752 10.1080/10618600.2017.1356325 PMC5920646

[18] Wolf JM Koopmeiners JS Vock DM. A permutation procedure to detect heterogeneous treatment effects in randomized clinical trials while controlling the type I error rate. Clin Trials. 2022;19:512–521. 35531765 10.1177/17407745221095855 PMC9529771

[19] Pak K Uno H Kim DH. Interpretability of cancer clinical trial results using restricted mean survival time as an alternative to the hazard ratio. JAMA Oncol. 2017;3:1692–1696. 28975263 10.1001/jamaoncol.2017.2797 PMC5824272

[20] Huang B Kuan PF. Comparison of the restricted mean survival time with the hazard ratio in superiority trials with a time-to-event end point. Pharm Stat. 2018;17:202–213. 29282880 10.1002/pst.1846

[21] Rice TW Adelstein DJ Chidel MA. Benefit of postoperative adjuvant chemoradiotherapy in locoregionally advanced esophageal carcinoma. J Thorac Cardiovasc Surg. 2003;126:1590–1596. 14666038 10.1016/s0022-5223(03)01025-0

[22] Macdonald JS Smalley SR Benedetti J. Chemoradiotherapy after surgery compared with surgery alone for adenocarcinoma of the stomach or gastroesophageal junction. N Engl J Med. 2001;345:725–730. 11547741 10.1056/NEJMoa010187

[23] Pasquer A Gronnier C Renaud F. Impact of adjuvant chemotherapy on patients with lymph node-positive esophageal cancer who are primarily treated with surgery. Ann Surg Oncol. 2015;22(Suppl 3):S1340–S1349. 26065869 10.1245/s10434-015-4658-1

[24] Deng X He W Jiang Y. The impact of adjuvant therapy on survival for node-negative esophageal squamous cell carcinoma: a propensity score-matched analysis. Ann Transl Med. 2021;9:998. 34277798 10.21037/atm-21-2539 PMC8267332

[25] Rice TW Lu M Ishwaran H; Worldwide Esophageal Cancer Collaboration Investigators. Precision surgical therapy for adenocarcinoma of the esophagus and esophagogastric junction. J Thorac Oncol. 2019;14:2164–2175. 31442498 10.1016/j.jtho.2019.08.004 PMC6876319

[26] Kelly RJ Ajani JA Kuzdzal J; CheckMate 577 Investigators. Adjuvant nivolumab in resected esophageal or gastroesophageal junction cancer. N Engl J Med. 2021;384:1191–1203. 33789008 10.1056/NEJMoa2032125

[27] Rice TW Zuccaro G Jr Adelstein DJ. Esophageal carcinoma: depth of tumor invasion is predictive of regional lymph node status. Ann Thorac Surg. 1998;65:787–792. 9527214 10.1016/s0003-4975(97)01387-8

[28] Riquet M Saab M Le Pimpec Barthes F. Lymphatic drainage of the esophagus in the adult. Surg Radiol Anat. 1993;15:209–211. 8235965 10.1007/BF01627708

[29] Kuge K Murakami G Mizobuchi S. Submucosal territory of the direct lymphatic drainage system to the thoracic duct in the human esophagus. J Thorac Cardiovasc Surg. 2003;125:1343–1349. 12830054 10.1016/s0022-5223(03)00036-9

[30] Murakami G Sato I Shimada K. Direct lymphatic drainage from the esophagus into the thoracic duct. Surg Radiol Anat. 1994;16:399–407. 7725196 10.1007/BF01627660

[31] van Leeuwen PJ Kranse R Hakulinen T. Disease-specific mortality may underestimate the total effect of prostate cancer screening. J Med Screen. 2010;17:204–210. 21258131 10.1258/jms.2010.010074

[32] Black WC Haggstrom DA Welch HG. All-cause mortality in randomized trials of cancer screening. J Natl Cancer Inst. 2002;94:167–173. 11830606 10.1093/jnci/94.3.167

[33] Markar SR Gronnier C Pasquer A; FREGAT working group – FRENCH – AFC. Role of neoadjuvant treatment in clinical T2N0M0 oesophageal cancer: results from a retrospective multi-center European study. Eur J Cancer. 2016;56:59–68. 26808298 10.1016/j.ejca.2015.11.024

[34] Goense L Visser E Haj Mohammad N. Role of neoadjuvant chemoradiotherapy in clinical T2N0M0 esophageal cancer: a population-based cohort study. Eur J Surg Oncol. 2018;44:620–625. 29478739 10.1016/j.ejso.2018.02.005

