# Supplementary material for: Adjuvant Therapy after Esophagectomy for Esophageal Cancer: Who Needs It?: Multi-institution Worldwide Observational Study
Source: Ann Surg Open. 2024 Oct 15;5(4):e497. doi: 10.1097/AS9.0000000000000497 (PMC11661710; doi:10.1097/AS9.0000000000000497)
Supplement: Supplementary file 1 [file as9-5-e497-s001.pdf]

Table S1. Worldwide Esophageal Cancer Collaboration: Participating Institutions and Investigators

Institution	Location	Investigators
Beijing Cancer Hospital, Peking University	Beijing, China	Ken N. Chen
Cleveland Clinic	Cleveland, OH; USA	Thomas W. Rice<br>Eugene H. Blackstone
Case Western Reserve University	Cleveland, OH; USA	Carolyn Apperson-Hansen
Erasmus Medical Center	Rotterdam, The Netherlands	Bas P.L. Wijnhoven<br>Jan van Lanschot<br>Sjoerd Lagarde
Fourth Hospital of Hebei Medical University	Shijiazhuang, Hebei; China	Jun-Feng Liu
Fox Chase Cancer Center	Philadelphia, PA; USA	Walter J. Scott<br>Donna Edmondson
Groote Schuur Hospital, University of Cape Town	Cape Town, South Africa	Riette Burger
Guy’s & St. Thomas’ Hospitals	London, UK	Andrew R. Davies<br>Janine Zylstra
Helsinki University Hospital	Helsinki, Finland	Jari V. Räsänen<br>Jarmo A. Salo<br>Yvonne Sundstrom
Hospital Universitario del Mar	Barcelona, Spain	Manuel Pera
Hôpital Nord	Marseille, France	Xavier B. D'Journo
Indiana University Medical Center	Indianapolis, IN; USA	Kenneth A. Kesler
University of Texas MD Anderson Hospital	Houston, TX; USA	Wayne L. Hofstetter<br>Arlene Correa<br>Stephen G. Swisher
Mayo Clinic	Rochester, MN; USA	Mark S. Allen
Medical University of South Carolina	Charleston, SC; USA	Chad E. Denlinger
Memorial Sloan-Kettering Cancer Center	New York, NY; USA	Valerie W. Rusch
University of Queensland, Princess Alexandra Hospital	Brisbane, Australia	B. Mark Smithers<br>David Gotley<br>Andrew Barbour<br>Iain Thomson
University of Newcastle upon Tyne	Newcastle upon Tyne, UK	S. Michael Griffin<br>Jon Shenfine

Oregon Health & Science University	Portland, OR; USA	Paul H. Schipper<br>John G. Hunter
Royal Marsden NHS Foundation Trust	London, UK	William H. Allum
Shanghai Chest Hospital	Shanghai, China	Wentao (Vincent) Fang
Toronto General Hospital	Toronto, ON; Canada	Gail E. Darling
University Zeikenhuizen Leuven	Leuven, Belgium	Tony E.M.R. Lerut<br>Phillipe R. Nafteux
University Medical Center Utrecht	Utrech, The Netherlands	Richard van Hillegersberg
University of Alabama at Birmingham	Birmingham, AL; USA	Robert J. Cerfolio
Hospital de Clinicas,<br>University of Buenos Aires	Buenos Aires, Argentina	Luis Durand<br>Roberto De Antón
The University of Chicago, Department of Surgery	Chicago, IL; USA	Mark K. Ferguson
University of Hong Kong Medical Center, Queen Mary Hospital	Hong Kong, China	Simon Law
University of Michigan	Ann Arbor, MI; USA	Mark B. Orringer<br>Becky L. Marshall
University of Montreal	Montreal, Quebec; Canada	André Duranceau<br>Susan Howson
University of Pittsburgh Medical Center	Pittsburgh, PA; USA	James D. Luketich<br>Arjun Pennathur<br>Kathy Lovas
University of Rochester	Rochester, NY; USA	Thomas J. Watson
University of São Paulo	São Paulo, Brazil	Ivan Cecconello
West China Hospital of Sichuan University	Chengdu, Sichuan; China	Long-Qi Chen

Table S2. Baseline characteristics and treatment of patients undergoing esophagectomy alone or combined with adjuvant therapy for cancer of the esophagus and esophagogastric junction

Variables	Esophagectomy Alone (N=10,873)		Adjuvant Therapy after Esophagectomy (N=2278)	
	n[a]	No (%) or Mean±SD	n[a]	No (%) or Mean±SD
Demographics				
Age (years)	10,571	63 ± 11	2278	59 ± 9.3
Female	10,871	2328 (21)	2278	341 (15)
Body mass index (kg/m^2)	5811	25 ± 5.1	1485	23 ± 3.9
Weight loss within 3 months before treatment (kg)	5982	1.9 ± 8.9	1655	1.6 ± 4.5
Clinical status				
ECOG score	2517		1222	
0		613 (24)		300 (25)
1		680 (27)		181 (15)
2		827 (33)		529 (43)
3		386 (15)		208 (17)
4		11 (0.44)		4 (0.33)
Comorbidities				
Diabetes	9155	808 (8.8)	1910	97 (5.1)
Insulin dependent	808	125 (15)	97	21 (21)
Non–insulin dependent	808	405 (50)	97	52 (54)
Not specified	808	278 (34)	97	24 (25)
Coronary artery disease	3979	525 (13)	1367	47 (3.4)
Atrial arrhythmia	3320	82 (2.5)	1262	21 (1.7)
Hypertension	7063	1829 (26)	1797	295 (16)
Peripheral artery disease	4989	180 (3.6)	1557	21 (1.3)
Smoking history	7077	4897 (69)	1697	1206 (71)
Past	4897	1904 (39)	1206	516 (43)
Current	4897	1620 (33)	1206	569 (47)
Not specified	4897	1373 (28)	1206	121 (10)

FEV1 (%)	4554	96 ± 21	1370	99 ± 20
Creatinine (mg/dL)	1965	74 ± 24	1104	77 ± 16
Bilirubin (mg/dL)	1735	12 ± 6.7	1119	12 ± 5.7
Adjuvant therapy	—		2278	
Chemotherapy		—		1049 (46)
Radiotherapy		—		381 (17)
Chemoradiotherapy		—		819 (36)
Not specified		—		29 (1.3)
Histopathologic	10,873		2278	
Adenocarcinoma		6560 (60)		966 (42)
Squamous cell carcinoma		4313 (40)		1312 (58)
Esophagectomy approach	10,873		2278	
Total MIE		467 (4.3)		24 (1.1)
Hybrid MIE		447 (4.1)		46 (2.0)
Thoracotomy/Transthoracic/ Ivor-Lewis		5626 (52)		1453 (64)
Thoracoabdominal		748 (6.9)		231 (10)
Transhiatal/Laparotomy		2311 (21)		288 (13)
Not stated		1274 (12)		236 (10)
Conduit	10,873		2278	
Stomach		8516 (78)		2037 (89)
Colon		169 (1.6)		33 (1.4)
Jejunum		250 (2.3)		25 (1.1)
Not stated		1938 (18)		183 (8.0)

a. Patients with data available.

Key: *ECOG*, Eastern Cooperative Oncology Group; *FEV1*, forced expiratory volume in 1 second (percent of predicted); *MIE*, minimally invasive esophagectomy; *SD*, standard deviation.

Table S3. Baseline characteristics and treatment of patients undergoing esophagectomy therapy for cancer of the esophagus and esophagogastric junction, stratified by histopathologic cell type

Variables	Adenocarcinoma (N=7526) n[a]	Adenocarcinoma (N=7526) No (%) or Mean±SD	Squamous Cell (N=5625) n[a]	Squamous Cell (N=5625) No (%) or Mean±SD
Demographics				
Age (years)	7258	64 ± 11	5591	60 ± 9.7
Female	7524	1114 (15)	5625	1555 (28)
Body mass index (kg/m^2)	3978	27 ± 5.0	3318	22 ± 3.4
Weight loss within 3 months before treatment (kg)	4168	2.2 ± 10	3469	1.5 ± 4.3
Clinical status				
ECOG score	1262		2477	
0		486 (39)		427 (17)
1		576 (46)		285 (12)
2		123 (9.7)		1233 (50)
3		68 (5.4)		526 (21)
4		9 (0.71)		6 (0.24)
Comorbidities				
Diabetes	5913	715 (12)	5152	190 (3.7)
Insulin dependent	715	111 (15)	190	35 (18)
Non–insulin dependent	715	340 (47)	190	117 (62)
Not specified	715	264 (37)	190	38 (20)
Coronary artery disease	2362	459 (19)	2984	113 (3.8)
Atrial arrhythmia	1750	64 (3.7)	2832	39 (1.4)
Hypertension	4923	1484 (30)	3937	640 (16)
Peripheral artery disease	3177	145 (4.6)	3369	56 (1.7)
Smoking history	5106	3523 (69)	3668	2580 (70)
Past	3523	1434 (41)	2580	986 (38)
Current	3523	982 (28)	2580	1207 (47)
Not specified	3523	1107 (31)	2580	387 (15)
FEV1 (%)	3053	95 ± 20	2871	98 ± 21

Creatinine (mg/dL)	755	73 ± 33	2314	76 ± 16
Bilirubin (mg/dL)	580	11 ± 6.2	2274	13 ± 6.3
Adjuvant therapy	7526	966 (13)	5625	1312 (23)
Chemotherapy	966	403 (42)	1312	646 (49)
Radiotherapy	966	128 (13)	1312	253 (19)
Chemoradiotherapy	966	406 (42)	1312	413 (31)
Not specified	966	29 (3.0)	1312	0 (0)
Esophagectomy approach	7526		5625	
Total MIE		399 (5.3)		92 (1.6)
Hybrid MIE		339 (4.5)		154 (2.7)
Thoracotomy/Transthoracic/ Ivor-Lewis		3031 (40)		4048 (72)
Thoracoabdominal		575 (7.6)		404 (7.2)
Transhiatal/Laparotomy		2139 (28)		460 (8.2)
Not stated		1043 (14)		467 (8.3)
Conduit	7526		5625	
Stomach		5565 (74)		4988 (89)
Colon		119 (1.6)		83 (1.5)
Jejunum		259 (3.4)		16 (0.28)
Not stated		1583 (21)		538 (9.6)

a. Patients with data available.

Key: *ECOG*, Eastern Cooperative Oncology Group; *FEV1*, forced expiratory volume in 1 second (percent of predicted); *MIE*, minimally invasive esophagectomy; *SD*, standard deviation.

Appendix S1. Variables Used in Random Forest Analyses

Demographics

Age (y), sex, race

Patient characteristics

Body mass index (kg/m^2), weight loss within 3 months before treatment, Eastern Cooperative Oncology Group performance status

Comorbidities

Diabetes, coronary artery disease, peripheral artery disease, arrhythmia, hypertension, smoking history, other cancers

Laboratory values

Forced expiratory volume in 1 second (percent of predicted), creatinine (mg/dL), bilirubin (mg/dL)

Pre-treatment cancer characteristics

Barrett esophagus, cT, cN, number of positive regional nodes, cM, histologic grade, location, distance from incisors to top of cancer, length of cancer

Pathologic cancer characteristics

pT, pN, number of positive regional lymph nodes, extracapsular lymph node invasion, lymphovascular invasion, pM, histologic grade

Resection

Number of lymph nodes resected, completeness of surgical resection (coded as R0, R1, or R2), margin positive

Appendix S2. Patient Eligibility (Overlap): Estimating the Probability of Therapy Received

In a randomized trial, the probability of receiving one or the other treatment is assigned at random. In observational studies such as this, therapy can depend on patient and cancer characteristics and other reasons not captured in the data. “Patient overlap” is a measure of the randomness of assigned treatment. It was evaluated by estimating the probability of treatment received according to patient and cancer characteristics. In the case of adjuvant therapy after esophagectomy, there was considerable imbalance in treatment received. The imbalanced ratio for adenocarcinoma was 6649/988 = 6.7 and for squamous cell carcinoma 4402/1334 = 3.3. To properly account for this imbalance, random forests quantile classification (RFQ) was used.[1] RFQ was implemented using default settings of randomForestSRC R-software.[2] Performance of the classifier was evaluated by geometric (G) mean (maximum value of 1 is perfect) and normalized Brier score (minimum value of 0 is perfect). For adenocarcinoma, G-mean was .82 for esophagectomy alone vs. adjuvant therapy after esophagectomy and .75 for squamous cell carcinoma. For normalized Brier score, the respective values were .29 and .54.

To deal with treatment overlap, we selected overlap cutoff values, restricting observations included in the analysis (eligibility) to patients with out-of-bag predicted values between 0.05 and 0.99.

References

1. O’Brien R, Ishwaran H. A random forests quantile classifier for class imbalanced data. *Pattern Recognit.* 2019;90:232-249.

2. Ishwaran H, Kogalur UB. Random forests for survival, regression and classification (RF-SRC), R package version 1.4.0.16. URL: http://cran.r-project.org/web/packages/randomForestSRC/index.html. 2014.

Table S4. Cancer characteristics of patients eligible for both esophagectomy alone or combined with adjuvant therapy for adenocarcinoma vs. non-exchangeability of treatments for patients with a low or high probability of receiving adjuvant therapy after esophagectomy

	Probability of Receiving Adjuvant Therapy after Esophagectomy					
	Low (excluded) (N=3775)		High (excluded) (N=48)		Included in study (N=3703)	
Cancer Characteristics	n[a]	No. (%)	n[a]	No. (%)	n[a]	No. (%)
Adjuvant therapy	3775	36 (0.95)	48	44 (92)	3703	886 (24)
Clinical T category	2626		27		2647	
0		129 (4.9)		0 (0)		19 (0.72)
Tis		173 (6.6)		0 (0)		33 (1.2)
1		971 (37)		0 (0)		303 (11)
2		568 (22)		2 (7.4)		720 (27)
3		742 (28)		25 (93)		1405 (53)
4		43 (1.6)		0 (0)		167 (6.3)
Pathologic T category	3768		48		3684	
Tis		376 (10)		0 (0)		34 (0.92)
1		1960 (52)		0 (0)		334 (9.1)
2		419 (11)		1 (2.1)		527 (14)
3		995 (26)		46 (96)		2563 (70)
4		18 (0.48)		1 (2.1)		226 (6.1)
Difference in T category	2620		27		2629	
Upstaged 4		0 (0)		0 (0)		13 (0.49)
Upstaged 3		3 (0.11)		0 (0)		24 (0.91)
Upstaged 2		120 (4.6)		0 (0)		158 (6.0)
Upstaged 1		314 (12)		3 (11)		592 (23)
Unchanged		1467 (56)		24 (89)		1429 (54)

Downstaged 1		476 (18)		0 (0)		318 (12)
Downstaged 2		188 (7.2)		0 (0)		78 (3.0)
Downstaged 3		51 (1.9)		0 (0)		16 (0.61)
Downstaged 4		1 (0.04)		0 (0)		1 (0.04)
Clinical cN+	2687	808 (30)	28	14 (50)	2652	1384 (52)
Pathologic pN+	3752	935 (25)	48	47 (98)	3678	2910 (79)
Clinical N category	1886		17		1471	
0		1879 (99.6)		14 (82)		1268 (86)
1		7 (0.37)		3 (18)		146 (9.9)
2		0 (0)		0 (0)		46 (3.1)
3		0 (0)		0 (0)		11 (0.75)
Pathologic N category	3556		43		3356	
0		2817 (79)		1 (2.3)		768 (23)
1		313 (8.8)		7 (16)		839 (25)
2		255 (7.2)		9 (21)		866 (26)
3		171 (4.8)		26 (60)		883 (26)
Difference in N	1878		17		1460	
Upstaged 3		46 (2.4)		5 (29)		230 (16)
Upstaged 2		90 (4.8)		6 (35)		325 (22)
Upstaged 1		115 (6.1)		5 (29)		409 (28)
Unchanged		1622 (86)		1 (5.9)		428 (29)
Downstaged 1		5 (0.27)		0 (0)		57 (3.9)
Downstaged 2		0 (0)		0 (0)		11 (0.75)
Resection margin	3775		48		3703	
0		3627 (96)		39 (81)		3094 (84)
1		109 (2.9)		9 (19)		478 (13)
2		39 (1.0)		0 (0)		131 (3.5)

a. Patients with data available.

Table S5. Cancer characteristics of patients eligible for both esophagectomy alone or combined with adjuvant therapy for squamous cell carcinoma vs. non-exchangeability of treatments for patients with a low or high probability of receiving adjuvant therapy after esophagectomy

	Probability of Receiving Adjuvant Therapy after Esophagectomy					
	Low (excluded) (N=1778)		High (excluded) (N=60)		Included in study (N=3787)	
**Cancer Characteristics**	**n[a]**	**No. (%)**	**n[a]**	**No. (%)**	**n[a]**	**No. (%)**
Adjuvant therapy	1778	25 (1.4)	60	58 (97)	3787	1229 (32)
Clinical T category	737		59		3341	
0		3 (0.41)		0 (0)		11 (0.33)
Tis		13 (1.8)		0 (0)		44 (1.3)
1		203 (28)		7 (12)		246 (7.4)
2		184 (25)		12 (20)		630 (19)
3		323 (44)		38 (64)		1634 (49)
4		11 (1.5)		2 (3.4)		776 (23)
Pathologic T category	1778		60		3785	
Tis		32 (1.8)		0 (0)		67 (1.8)
1		374 (21)		3 (5.0)		484 (13)
2		386 (22)		11 (18)		650 (17)
3		962 (54)		41 (68)		2021 (53)
4		24 (1.3)		5 (8.3)		563 (15)
Difference in T category	737		59		3340	
Upstaged 5		0 (0)		0 (0)		2 (0.06)
Upstaged 4		0 (0)		0 (0)		3 (0.09)
Upstaged 3		2 (0.27)		1 (1.7)		11 (0.33)
Upstaged 2		27 (3.7)		2 (3.4)		78 (2.3)
Upstaged 1		98 (13)		9 (15)		451 (14)

Unchanged		410 (56)		42 (71)		1796 (54)
Downstaged 1		146 (20)		5 (8.5)		761 (23)
Downstaged 2		48 (6.5)		0 (0)		196 (5.9)
Downstaged 3		6 (0.81)		0 (0)		41 (1.2)
Downstaged 4		0 (0)		0 (0)		1 (0.03)
Clinical cN+	756	303 (40)	58	32 (55)	3326	2201 (66)
Pathologic pN+	1776	422 (24)	60	59 (98)	3781	1944 (51)
Clinical N category (cN)	497		57		2790	
0		453 (91)		26 (46)		1125 (40)
1		44 (8.9)		29 (51)		1322 (47)
2		0 (0)		2 (3.5)		311 (11)
3		0 (0)		0 (0)		32 (1.1)
Pathologic N category	1748		59		3671	
0		1354 (77)		1 (1.7)		1837 (50)
1		308 (18)		40 (68)		989 (27)
2		81 (4.6)		18 (31)		623 (17)
3		5 (0.29)		0 (0)		222 (6.0)
Difference in N category	496		57		2783	
Upstaged 3		0 (0)		0 (0)		48 (1.7)
Upstaged 2		12 (2.4)		3 (5.3)		186 (6.7)
Upstaged 1		50 (10)		35 (61)		488 (18)
Unchanged		390 (79)		18 (32)		1134 (41)
Downstaged 1		44 (8.9)		0 (0)		771 (28)
Downstaged 2		0 (0)		1 (1.8)		138 (5.0)
Downstaged 3		0 (0)		0 (0)		18 (0.65)
Resection margin	1778		60		3787	
0		1688 (95)		60 (100)		3487 (92)
1		87 (4.9)		0 (0)		229 (6.0)
2		3 (0.17)		0 (0)		71 (1.9)

a. Patients with data available.

Appendix S3. Survival Analysis

Individual treatment effect was estimated using random survival forests virtual-twin interaction RSF-VT-I.[1] For each patient, RSF-VT-I yielded an out-of-sample survival estimate as well as predicted survival if the patient had received the counterfactual (alternative) treatment to what he or she actually received. Augmented virtual-twin data matrices contained 239 variables, and random feature selection used 200 variables. Survival trees used log-rank splitting, in which each tree was calculated using a subsample without replacement of size 63.2% of original data. Each analysis used 1000 trees.

Reference

1. Lu M, Sadiq S, Feaster DJ, et al. Estimating individual treatment effect in observational data using random forest methods. *J Comput Graph Stat.* 2018;27:209-219.

Table S6. Baseline characteristics of patients deemed eligible for both esophagectomy alone or combined with adjuvant therapy, stratified by pure histopathologic cell type

	Esophagectomy Alone				Adjuvant Therapy after Esophagectomy			
	Adenocarcinoma (N=2817)		Squamous Cell Carcinoma (N=2558)		Adenocarcinoma (N=886)		Squamous Cell Carcinoma (N=1229)	
**Baseline Characteristics**	**n[a]**	**No. (%) or Mean ± SD**	**n[a]**	**No. (%) or Mean ± SD**	**n[a]**	**No. (%) or Mean ± SD**	**n[a]**	**No. (%) or Mean ± SD**
Demographics								
Age (y)	2719	64 ± 11	2534	62 ± 9.2	886	60 ± 9.9	1229	59 ± 8.5
Female	2815	410 (15)	2558	661 (26)	886	96 (11)	1229	228 (19)
Body mass index (kg/m^2)	1614	26 ± 5.0	1850	22 ± 3.5	446	25 ± 4.7	1013	22 ± 3.0
Patient weight loss within 3 months before treatment (kg)	1617	3.0 ± 11	1929	1.8 ± 4.6	544	1.7 ± 5.8	1059	1.6 ± 3.3
Clinical status								
ECOG score	480		1447		242		956	
0		171 (36)		217 (15)		101 (42)		186 (19)
1		189 (39)		164 (11)		84 (35)		89 (9.3)
2		75 (16)		729 (50)		33 (14)		493 (52)
3		41 (8.5)		334 (23)		22 (9.1)		186 (19)
4		4 (0.83)		3 (0.21)		2 (0.83)		2 (0.21)

Clinical T category (cT)	2012		2190		635		1151	
0		14 (0.70)		11 (0.50)		5 (0.79)		0 (0)
Tis		28 (1.4)		38 (1.7)		5 (0.79)		6 (0.52)
1		261 (13)		207 (9.5)		42 (6.6)		39 (3.4)
2		560 (28)		437 (20)		160 (25)		193 (17)
3		1033 (51)		1038 (47)		372 (59)		596 (52)
4		116 (5.8)		459 (21)		51 (8.0)		317 (28)
X		805		368		251		78
Clinical N category (cN)	1124		1732		347		1058	
0		1025 (91)		796 (46)		243 (70)		329 (31)
1		77 (6.9)		752 (43)		69 (20)		570 (54)
2		16 (1.4)		172 (9.9)		30 (8.6)		139 (13)
3		6 (0.53)		12 (0.69)		5 (1.4)		20 (1.9)
X		1693		826		539		171
Comorbidities								
Diabetes	2356	305 (13)	2304	99 (4.3)	610	54 (8.9)	1181	37 (3.1)
Insulin dependent	305	49 (16)	99	14 (14)	54	12 (22)	37	9 (24)
Non–insulin dependent	305	141 (46)	99	67 (68)	54	30 (56)	37	21 (57)
Not stated	305	115 (38)	99	18 (18)	54	12 (22)	37	7 (19)
Coronary artery disease	925	213 (23)	1615	74 (4.6)	248	22 (8.9)	1035	23 (2.2)
Atrial arrhythmia	752	33 (4.4)	1531	24 (1.6)	184	9 (4.9)	1004	12 (1.2)
Hypertension	1979	582 (29)	2070	344 (17)	550	153 (28)	1143	129 (11)
Peripheral artery disease	1374	78 (5.7)	1825	25 (1.4)	384	12 (3.1)	1081	8 (0.74)

Smoking history	1538	1401 (71)	1856	1446 (71)	497	425 (73)	1031	748 (71)
Past	1401	541 (39)	1446	596 (41)	338	202 (48)	748	299 (40)
Current	1401	437 (31)	1446	672 (46)	338	136 (32)	748	422 (56)
Not Stated	1401	423 (30)	1268	178 (12)	338	87 (20)	748	27 (3.6)
FEV1 (% of predicted)	1133	92 ± 21	1595	99 ± 22	415	97 ± 19	920	101 ± 20
Creatinine (mg/dL)	337	74 ± 32	1364	75 ± 17	179	78 ± 18	912	77 ± 15
Bilirubin (mg/dL)	232	11 ± 5.9	1333	13 ± 6.8	192	12 ± 5.9	913	12 ± 5.6
Adjuvant therapy	—	—	—	—				
Chemotherapy only	—	—	—	—	886	370 (41)	1229	582 (47)
Radiotherapy only	—	—	—	—	886	112 (13)	1229	243 (20)
Chemoradiotherapy	—	—	—	—	886	378 (43)	1229	404 (33)
Not stated	—	—	—	—	886	26 (2.9)		0 (0)
Esophagectomy approach	2817		2558		886		1229	
Total MIE		111 (3.9)		53 (2.1)		20 (2.3)		2 (0.16)
Hybrid MIE		129 (4.6)		73 (2.9)		8 (0.90)		33 (2.7)
Thoracotomy/Transthoracic/ Ivor-Lewis		1305 (46)		1693 (66)		373 (42)		993 (81)
Thoracoabdominal		211 (7.5)		224 (8.8)		121 (14)		95 (7.7)
Transhiatal/Laparotomy		695 (25)		263 (10)		195 (22)		66 (5.4)
Not stated		366 (13)		252 (9.9)		169 (19)		40 (3.3)

Conduit	2817	2558	886	1229
Stomach	2103 (75)	2206 (86)	706 (80)	1193 (97)
Colon	46 (1.6)	44 (1.7)	21 (2.4)	11 (0.90)
Jejunum	132 (4.7)	13 (0.51)	24 (2.7)	1 (0.08)
Not stated	536 (19)	295 (12)	135 (15)	24 (2.0)

a. Patients with data available.

Key: *ECOG*, Eastern Cooperative Oncology Group; *FEV1*, forced expiratory volume in 1 second; *MIE*, minimally invasive esophagectomy; *SD*, standard deviation.

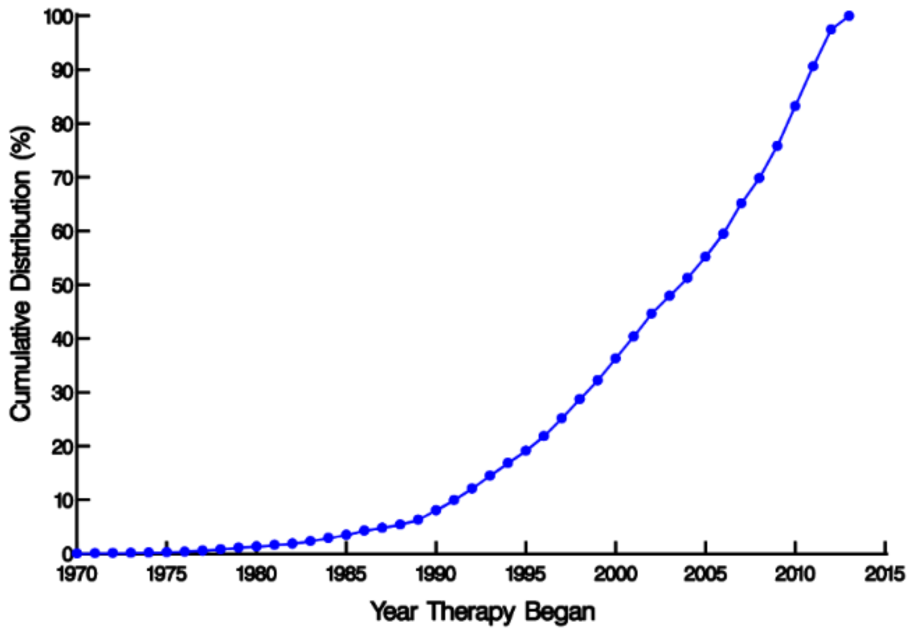

Figure S1: Cumulative distribution of date therapy began among patients eligible for the virtual-twin comparative analysis.
